# Professionalism in Family Planning Care Workshop

**DOI:** 10.15766/mep_2374-8265.11212

**Published:** 2022-01-12

**Authors:** Jody Steinauer, Aliza Adler, Jema Turk, Jessie Chien, Uta Landy

**Affiliations:** 1 Distinguished Professor, Department of Obstetrics, Gynecology, and Reproductive Sciences, University of California, San Francisco, School of Medicine; 2 Program and Academic Coordinator, Department of Obstetrics, Gynecology, and Reproductive Sciences, University of California, San Francisco, School of Medicine; 3 Director, Ryan Program, Department of Obstetrics, Gynecology, and Reproductive Sciences, University of California, San Francisco, School of Medicine; 4 PhD Candidate, Department of Community Health Sciences, University of California, Los Angeles, Fielding School of Public Health; 5 Senior Advisor, Department of Obstetrics, Gynecology, and Reproductive Sciences, University of California, San Francisco, School of Medicine

**Keywords:** Professionalism, Family Planning, Women's Health, OB/GYN

## Abstract

**Introduction:**

When clinicians feel negative emotions toward patients, providinge patient-centered care can be difficult. This can occur in family planning scenarios, such as when a provider is uncomfortable with a patient choosing abortion. The Professionalism in Family Planning Care Workshop (PFPCW), framed around professionalism values, used guided reflection to foster self-awareness and empathy in order to teach future providers to provide patient-centered care.

**Methods:**

In the PFPCW, learners discussed challenging patient interactions and family planning scenarios to develop self-awareness and identify strategies for maintaining therapeutic relationships with patients when they experience negative feelings toward them. We implemented the workshop across the United States and Canada and collected pre- and postsurvey data to evaluate program outcomes at Kirkpatrick evaluation levels of participant reaction and effects on learners’ attitudes.

**Results:**

A total of 403 participants participated in 27 workshops in which pre- and postworkshop surveys (70% and 46% response rates, respectively) were administered. Sixty-five percent of the participants were residents, and 36% had previously participated in a similar workshop. The majority (92%) rated the PFPCWs as worthwhile. Participants valued the discussion and self-reflection components. Afterward, 23% reported that their attitudes toward caring for people with unintended pregnancy changed to feeling more comfortable. Participants said they would employ self-reflection and empathy in future challenging interactions.

**Discussion:**

In this pilot implementation study, our workshop provided learners with strategies for patient-centered care in challenging family planning patient interactions. We are currently modifying the workshop and evaluation program based on feedback.

## Educational Objectives

By the end of this activity, workshop participants will be able to:

1. Reflect on interactions in which they felt negative emotions toward patients.
2. Reflect on their feelings about family planning clinical care scenarios.
3. Discuss strategies, such as understanding the patient context and finding empathy, to help provide high-quality care for patients who make decisions about health care with which the provider may disagree.

## Introduction

Clinicians’ negative feelings, such as frustration or anger, toward patients can make it challenging to provide compassionate, high-quality care based on the profession's values of patient-centeredness, patient autonomy, and primacy of patient welfare.^1^ Clinicians’ negative emotions are sometimes triggered in common family planning scenarios, such as a patient facing an undesired pregnancy or desiring an abortion.^2–4^ Furthermore, providers may encounter unintentional feelings of discomfort and judgment toward patients’ pregnancy and contraceptive decision-making, which can lead to frustration and result in lower-quality patient care.^5^

Due to the prevalence of contraceptive use and abortion incidence, it is important for clinicians in primary care or obstetrics and gynecology (OB/GYN) to develop self-awareness of personal beliefs about contraception and pregnancy decision-making, including abortion, adoption, and parenting, and to cultivate strategies to provide compassionate care regardless of personal beliefs.^6,7^ Clinicians are required to provide objective, supportive pregnancy options and contraceptive counseling and referral, as well as contraceptive and abortion care, to their level of conscience and skill. Providing the highest quality of care in these interactions is particularly salient in light of a long-standing history of coercive contraception and sterilization practices experienced by Black, Hispanic, and Native American women, particularly those of lower education and income, in the United States.^8^

Health professions educators must support learners in cultivating patient-centered care skills, as learners are more likely to experience negative emotions toward patients than practicing clinicians.^9,10^ To meet undergraduate and graduate medical learners’ needs, the Ryan Residency Training Program, a national initiative that supports OB/GYN departments in formally integrating family planning in residency training in accordance with ACGME requirements, developed the Professionalism in Family Planning Care Workshop (PFPCW) geared toward OB/GYN residents. The PFPCW is based on a previously published workshop^11^ and integrates aspects of values clarification (VC) workshops.^12–14^ While VC is commonly used as a technique to support patients’ decision-making around health care decisions, VC workshops were designed to support family planning providers in exploring their own values and attitudes about contraception and pregnancy decision-making in order to improve the care they provided to family planning patients. VC workshops have been found to improve knowledge about and make attitudes more positive toward abortion as a patient's choice and to increase intentions to provide abortion care.^14^

**Figure 1.**
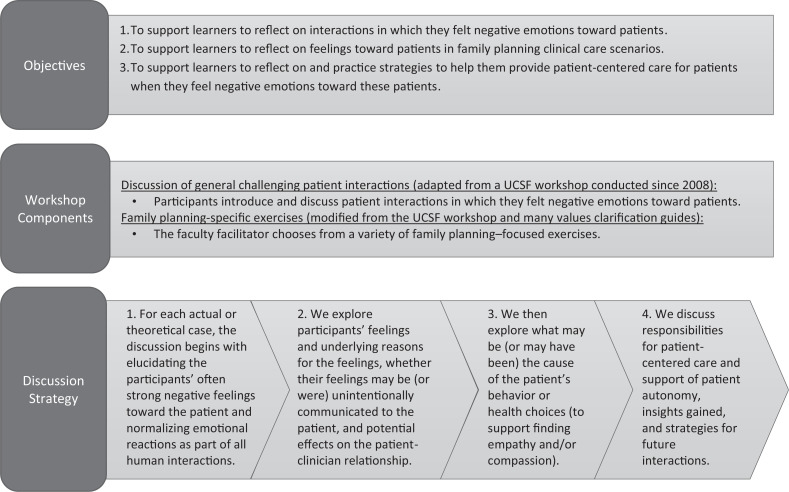
Description of the Professionalism in Family Planning Care Workshop. Abbreviation: UCSF, University of California, San Francisco.

While *MedEdPORTAL* publishes many resources on professionalism tools, there are limited curricula for maintaining professionalism with challenging patients in reproductive health–specific scenarios. Our curriculum builds on a previously published workshop^11^ in *MedEdPORTAL* by incorporating new emotionally challenging patient scenarios in family planning–specific situations and VC techniques, including a comprehensive facilitation guide. The previously published workshop was not family planning specific and was not framed around professionalism as the core value. The PFPCW objectives are to provide learners with the opportunity to reflect on their negative feelings toward patients, specifically in the context in family planning care, and to identify patient-centered care strategies (Figure 1). Our goal here is to describe the workshop, its implementation, and its outcomes, as conducted in OB/GYN departments in the United States and Canada from 2016 to 2019.

Beginning in 2016, the Ryan Program developed this workshop to support learners in reflecting on their feelings about patients’ family planning decisions and developing strategies to provide care consistent with medicine's professionalism principles. These principles include patient-centered care, which has been defined as “putting the patient at the heart of care delivery and working in partnership with the patient to ensure patients are well informed and their care choices are respected,”^15,16^ in addition to patient autonomy and the primacy of patient welfare. The workshop's strategies are based on the value of reflection in health professions education. The PFPCW was designed for OB/GYN residents, but workshops have included medical students and advanced-practice clinician learners as well.^17,18^

## Methods

### Workshop Development

**Figure 2.**
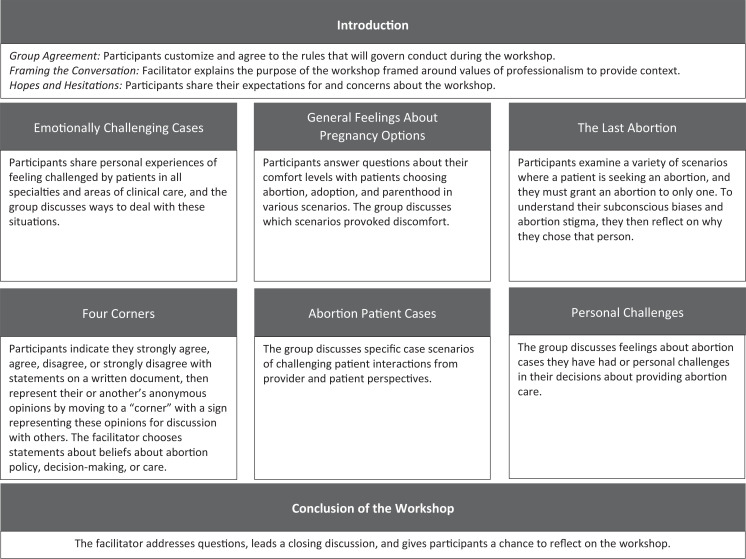
Components of the Professionalism in Family Planning Care Workshop.

The Ryan Program's advisory team built on the previously published Caring for Challenging Patients Workshop^11^ by integrating family planning exercises from published VC workshops^12–14^ to develop the PFPCW. The updated workshop included family planning–specific exercises and was framed around professionalism. The PFPCW had three introductory components and one concluding component as well as exercises that faculty members could choose from to tailor the workshop to meet specific learners’ needs: an exercise focused on discussing experiences with challenging patients in general and five exercises focused on family planning and abortion cases (Figure 2; Appendices A and B). Because the workshop was developed by the Ryan Program, the material primarily focused on family planning–related scenarios.

### Facilitator Training

When we launched the curriculum, we hosted a train-the-trainer (TTT) session for faculty to attend before facilitating any PFPCWs. Since that time, additional faculty have facilitated workshops after watching an online training video, reviewing the facilitation guide, and/or doing a one-on-one training with faculty trainers. The initial 5-hour, in-person TTT, conducted in 2015, included reviewing all workshop components, practicing facilitation skills within emotionally challenging conversations, and providing tools to guide learner reflections.

### Workshop Outline and Implementation

From 2016 to 2019, 13 trained faculty led workshops at 23 different institutions across the United States. Institutions were informed about the workshop by LISTSERV emails and scheduled workshops into their resident didactic sessions. Starting in 2020, as a result of the COVID-19 pandemic, all workshops were converted to a virtual format. The Ryan Program administered an evaluation, consisting initially of postworkshop surveys and then pre- and postworkshop surveys for participants (Appendices C and D) and postworkshop surveys for facilitators (Appendix E), to assess the PFPCW at levels 1 and 2 of the commonly used education evaluation framework designed by Kirkpatrick and colleagues.^19^ Kirkpatrick level 1 is evaluation of the learners’ experiences of and reactions to the workshop, and Kirkpatrick level 2 is the effect on learners’ skills, attitudes, beliefs, and intentions.

Before leading a PFPCW, facilitators received the PFPCW facilitation guide, draft agendas, pre- and postworkshop surveys to circulate to participants, and an informational video (Appendices A–F). The facilitation guide was intended to be used by facilitators as a preparation and planning tool for their workshop and to help walk them through each workshop component described below (Appendices B and C). Additionally, the guide included suggestions for preworkshop readings^20–23^ and exercises that could be sent to participants (Appendix B). Draft agendas (Appendix A) could be adapted by facilitators depending on exercises chosen and institution-specific details and were intended to be sent to participants ahead of the workshop.

Every PFPCW began with establishing a group agreement about rules to govern conduct during the workshop. Workshops typically lasted 1–3 hours, with 1 hour as the minimum suggested allotted time, and consisted of an introduction, chosen exercises, and a concluding discussion and guided reflection (Figures 1 and 2). Guided reflection was considered a critical component of experiential learning.^17,18^ Each facilitator chose which exercises to facilitate in the workshop from those listed in the workshop guide (Appendix B), and this determined what tools they needed, which might include whiteboards or printed handouts and, if done virtually, chat boxes and surveys. Facilitators utilized draft agendas (Appendix A) for guidance on structuring the workshop components.

For each clinical case, participants discussed their actual or potential negative feelings toward the patient, which normalized emotional reactions as common aspects of human interactions. We then explored participants’ feelings and underlying values about the case, whether their feelings might be (or were) unintentionally communicated to the patient and might affect (or did affect) the patient-doctor relationship and the care provided, what might cause (or had caused) the patient's behavior or health choices, and how participants would ideally provide patient-centered care with a similar patient in the future.

### Evaluation

The pre- and postworkshop participant surveys each consisted of 19 questions, with many subquestions (Appendices C and D). At baseline, participants reported their learner status, past participation in similar workshops, and past participation in abortion-related care in specific procedures and situations. These surveys were developed in conjunction with the PFPCW and utilized scales to measure the following variables. We assessed whether baseline empathy, psychosocial orientation, attitudes, religiosity, and religious affiliation correlated with participants’ experience of the workshop or workshop outcomes. Some of our evaluation measures (Appendices C–E) that had originally been crafted by the Ryan Residency Training Program National Office Staff were used to measure outcomes of a separate VC workshop.^24^

After the workshop, to evaluate level 1 outcomes, we asked participants to rate the value of the overall workshop and its individual parts on a 5-point Likert scale from extremely useless to extremely useful. Participants also described, in open-ended comments, what they liked most and least about the workshop and whether they felt comfortable sharing thoughts with the group. We examined whether there was an association between attitudinal factors and religiosity to the overall experience of the workshop and the experiences of specific components.

We compared responses to four case scenarios pre- and postworkshop. Before and after the workshop, learners reacted to four cases in which a woman was having or had just had an abortion. For each case, they indicated level of agreement (on a 5-point Likert scale from strongly disagree to strongly agree) with the following statements:

1. “I can think of a justifiable reason that would explain why the patient is in this circumstance and makes this decision.”
2. “This case makes me feel frustrated.”
3. “My reaction to this case would make it hard for me to care for the patient.”

Participants also reported (on a 5-point Likert scale from certainly no to certainly yes, with an “I don't know” option) their intention to provide abortion services in the future. Participants reported whether the workshop had affected their feelings about caring for women with unintended pregnancy (yes/no) and, if yes, explained how. We also asked them to think of a recent patient interaction in which they would have been able to use the strategies they had learned in this workshop and how, if at all, they would use the strategies they had practiced.

We analyzed open-ended responses using content analysis. Two authors (Jody Steinauer and Aliza Adler) conducted content analyses^25^ separately and reviewed each other's analysis to make sure they were in agreement.

### Statistical Analyses

We conducted descriptive statistics for learner characteristics and workshop outcomes. Responses to case scenarios were reported as agree, disagree, and neutral. For the empathy, physician belief, and abortion attitude scales, items were summed and left as continuous variables for analyses. We performed Fisher exact tests, Wilcoxon rank sum tests, and *t* tests to assess correlations between religiosity and attitudinal factors with usefulness of the workshop. To assess whether the workshop changed learners’ feelings toward patients in specific family planning circumstances, we fit mixed-effects logistic regression models for each of these outcomes to describe changes in the outcome pre- and postworkshop and controlling for attitudinal factors. We performed statistical analyses using Stata IC 15.1. We considered associations statistically significant at *p* < .05. The study was considered exempt by the University of California, San Francisco, Institutional Review Board.

## Results

From 2016 to 2019, in response to requests from OB/GYN departments, 13 faculty (11 OB/GYN faculty, one nurse-midwife faculty, and one independent health care consultant) conducted 27 workshops with a total of 403 participants, with a median of 16 learners per workshop. Institutions sent requests for workshops to the Ryan Program, and all requests were met. These 27 workshops took place at 23 unique institutions throughout the United States (five in the West, three in the Midwest, four in the South, and 10 in the Northeast) and one in Canada. Of these 27 workshops, evaluation data were collected at 22, with 339 participants, conducted by 10 trained faculty.

**Figure 3.**
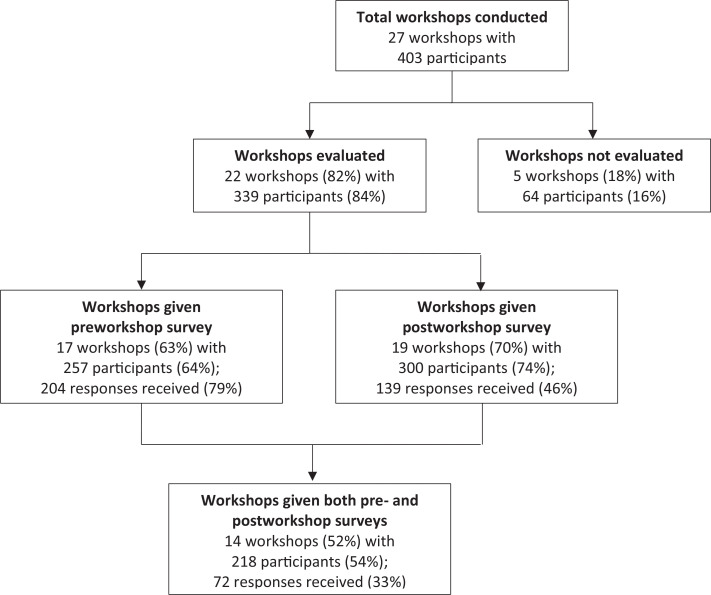
Professionalism in Family Planning Care Workshop evaluation survey distribution, 2016–2019. Proportions are out of the total of 27 workshops conducted and out of the total of 403 participants who attended. Survey response rates are calculated out of the total number of participants who received each survey.

A total of 257 learners participated in workshops where surveys were conducted before the workshop, with 204 (79%) responding; 300 participated in workshops where postworkshop surveys were collected, with 139 (46%) responding; and 218 participated in workshops with both surveys collected, with 72 (33%) completing both and able to be matched (Figure 3). Most workshops included the Four Corners exercise (85%), discussions of general challenging cases (70%) and of personal challenges with abortion care (70%), and review of specific abortion patient cases (65%).

### Baseline Data About Participants

The majority of participants (65%) were residents; medical students, family planning fellows, faculty physicians, nurses, and others also participated. At baseline, 36% had previously participated in similar workshops, and 83% reported that they felt comfortable sharing in groups where others had different opinions. Of the residents, 120 (92%) had past training in abortion care.

Before the workshop, in responding to the case scenarios, participants on average agreed that they could “think of justifiable reasons that would explain why this patient… makes this decision” and disagreed with the statements “This case makes me frustrated” and “My reaction to this case would make it hard for me to care for this patient.” However, some individuals were neutral or held the opposite opinions. Baseline reactions to the case scenarios among all participants are shown in the Table.

**Table.**
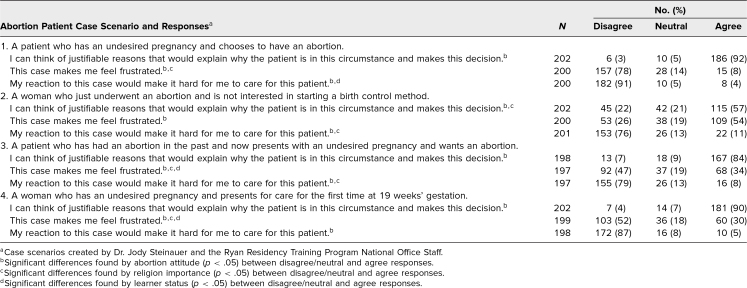
Workshop Participants’ Perceptions of Case Scenarios at Baseline

### Kirkpatrick Level 1 Outcomes: Participants’ Evaluation of Workshop

The majority of participants (92%) rated the PFPCW as useful. Ninety-five percent reported feeling comfortable and 5% uncomfortable sharing their feelings, and 99% of the latter said the level of comfort or discomfort was tolerable.

Across all participants, there were no differences in overall usefulness of the workshop and with specific components by religiosity and baseline abortion attitude and empathy. However, participants with lower comfort with psychosocial aspects of medicine scored three workshop components as more valuable. These included general feelings about pregnancy options, challenging cases, and personal challenges (*p*s = .04, .02, and .02, respectively).

When asked in open-ended questions what they liked most about the workshop, many participants cited the open discussion format and having a space to hear their colleagues’ perspectives, as well as the self-reflection components. When asked what they liked least, participants most frequently said the workshop was too short or detailed other logistical aspects of the workshop, including wanting to discuss more scenarios and disliking the specific Last Abortion exercise. A few participants felt there was not enough diversity of opinions expressed in the workshop because most participants identified as pro-choice; one described their workshop as an “echo chamber.”

### Kirkpatrick Level 2 Learning: Changes in Attitudes and Intentions

Comparing pre- and postworkshop responses, there was an increase in participants’ abilities to think of reasons why a postabortion patient would not be interested in starting a contraceptive method (62% vs. 93%, *p* < .01). Additionally, there was a small significant increase in the proportion who agreed that their reaction to a patient with a prior abortion presenting for another abortion would make it hard to care for this patient (1% vs. 7%, *p* = .04). There were no other statistically significant differences in other case attitude questions.

Participants at baseline were overall supportive of and comfortable with people seeking abortion care, but after the workshop, 23% indicated that their feelings about caring for women with unintended pregnancy had changed toward greater comfort. In open-ended comments, most participants stated that after the workshop, they felt more tolerant and open to patients’ reasons for terminating a pregnancy and generally described improved awareness of their feelings and understanding of reasons for abortion. Participants gave examples such as being more open to abortion than previously and not judging patients for having more than one abortion. Some expressed that the workshop helped them understand their personal feelings regarding family planning care, with one saying it “made me more excited to do this work.”

When asked if they could have used the learned strategies in the past or would in the future, participants referenced techniques of self-reflection and mindfulness with patients whose beliefs differed from theirs (e.g., choosing condoms or abortion as birth control). Additionally, they would focus on patients’ perspectives and practice empathy and open-mindedness in patient counseling. Another subset of participants reported that they would use the newly learned tools to practice self-reflection to recognize their biases in interactions with patients.^3,26^

## Discussion

Overall, participants found the PFPCW a useful tool for increasing empathy toward patients. Our data support that the workshop changed participants’ feelings and ability to find empathy. Participants noted several self-reflection and other tools they would use in future patient interactions, suggesting the workshop will aid them in future clinical encounters.

The data we collected support the importance of guided reflection about family planning clinical care. At baseline, while most participants could think of justifiable reasons for the scenarios we presented, a majority of residents were frustrated with three of the cases, especially with the postabortion patient not interested in initiating a contraceptive method. Also of note, while only a few participants thought their frustration would get in the way of providing patient-centered care, it has been documented that patients do notice clinicians’ frustration and experience lower quality care as a result.^27^ We also found a small but significant increase in the proportion of participants who felt their reaction toward a patient having a second abortion might interfere with the care they provided, suggesting that their self-awareness of this potential had changed.

While the participants generally praised the workshop, they listed aspects that could be improved. Some wanted time to discuss a greater variety of challenging cases, and others felt there was not enough diversity of opinion. Because the majority of physicians in general and OB/GYN residents specifically identify as pro-choice and because the workshop is typically requested by a program that has fully integrated abortion training, it may be challenging to create an environment that feels inclusive for participants of all perspectives. In response to this feedback, we are doing more online and one-on-one faculty development to support creating a space in which diverse voices can be heard. Some participants indicated that they did not like the Last Abortion exercise. As a result, we are generally recommending integrating this exercise only with adequate time for in-depth discussions. Additionally, to make the workshop more inclusive of diversity, we have changed all patient scenarios to be gender inclusive.

Our evaluation had limitations. The workshops were administered with a variety of learners, including some with faculty participants, and by a variety of faculty facilitators. We encountered limitations common in other studies^17^ such as measuring short-term, self-reported outcomes. Some participants may have been especially motivated to complete the surveys due to their personal beliefs. Response rates may have been low because we relied on facilitators to disseminate the surveys and did not provide incentives for completion. Additionally, we had no way of tracking whether learners completed postworkshop surveys, preventing targeted follow-ups.

Participants valued the PFPCW as a reflection exercise, articulated improved self-awareness, and planned to use the skills in future patient care. We encourage medical educators to facilitate discussions with trainees about actual and theoretical patient interactions that evoke emotional responses in order to improve patient-centered care skills. In the context of OB/GYN care, it is important to facilitate these discussions about family planning scenarios to encourage patient-centered care in all interactions. We plan to continue supporting programs to integrate this workshop and are developing tools for virtual workshop facilitation that we will post on our website. We hope educators will consider facilitating the PFPCW in their didactics sessions or will develop a similar workshop to promote patient-centered care in all patient interactions.

## Appendices


Editable Agendas.docxPFPCW Guide.docxProfessionalism Learner Presurvey.docxProfessionalism Learner Postsurvey.docxProfessionalism Facilitator Postsurvey.docxPFPCW Facilitator Training Video.mp4
*All appendices are peer reviewed as integral parts of the Original Publication.*
