# Supplementary material for: Professionalism in Family Planning Care Workshop
Source: MedEdPORTAL. 2022 Jan 12;18:11212. doi: 10.15766/mep_2374-8265.11212 (PMC8752579; doi:10.15766/mep_2374-8265.11212)
Supplement: Supplementary file 1 — Editable Agendas.docxPFPCW Guide.docxProfessionalism Learner Presurvey.docxProfessionalism Learner Postsurvey.docxProfessionalism Facilitator Postsurvey.docxPFPCW Facilitator Training Video.mp4 [file mep_2374-8265.11212-s001.zip › A. Editable Agendas.docx]

**Professionalism in Family Planning Care Workshop Agenda**

[Facilitator Name Here]

**[Site Name Here]**

[Month Date], [Year]

[Start Time]-[End Time] **(1 Hour)**

**Agenda:**

9:00 - 9:10 Introductions

9:10 - 9:15 Group Agreements

9:15 - 9:20 Framing the Conversation

9:20 - 9:30 General Feelings About Pregnancy Options or The Last Abortion debrief

9:30 - 9:50 Challenging Cases Introduction or Abortion Patient Cases

9:50 - 10:00 Wrap Up

***Homework:*** *Review Group Agreements, relevant readings, General Feelings About Pregnancy Options or The Last Abortion*

**Professionalism in Family Planning Care Workshop Agenda**

[Facilitator Name Here]

**[Site Name Here]**

[Month Date], [Year]

[Start Time]-[End Time] **(1.5 Hours, A)**

**Agenda:**

9:00 - 9:10 Introductions

9:10 - 9:15 Group Agreements

9:15 - 9:20 Framing the Conversation

9:20 - 9:30 General Feelings About Pregnancy Options or The Last Abortion debrief

9:30 - 9:50 Four Corners

9:50 - 10:20 Challenging Cases Introduction or Abortion Patient Cases

10:20 - 10:30 Wrap Up

***Homework:*** *Review Group Agreements, relevant readings, General Feelings About Pregnancy Options or The Last Abortion*

**Professionalism in Family Planning Care Workshop Agenda**

[Facilitator Name Here]

**[Site Name Here]**

[Month Date], [Year]

[Start Time]-[End Time] **(1.5 Hours, B)**

**Agenda:**

9:00 - 9:10 Introductions

9:10 - 9:15 Group Agreements

9:15 - 9:20 Framing the Conversation

9:20 - 9:30 General Feelings About Pregnancy Options or The Last Abortion debrief

9:30 - 9:50 Four Corners

9:50 - 10:20 Abortion Patient Cases

10:20 - 10:30 Wrap Up

***Homework:*** *Review Group Agreements, relevant readings, General Feelings About Pregnancy Options or The Last Abortion*

**Professionalism in Family Planning Care Workshop Agenda**

[Facilitator Name Here]

**[Site Name Here]**

[Month Date], [Year]

[Start Time]-[End Time] **(2 Hours, A)**

**Agenda:**

9:00 - 9:10 Introductions

9:10 - 9:15 Group Agreements

9:15 - 9:20 Framing the Conversation

9:20 - 9:40 Hopes and Hesitations

9:40 - 9:55 The Last Abortion Debrief

9:55 - 10:15 Four Corners

10:15 - 10:45 General Feelings About Pregnancy Options

10:45 - 11:00 Wrap Up

***Homework:*** *Review Group Agreements, relevant readings, General Feelings About Pregnancy Options or The Last Abortion*

**Professionalism in Family Planning Care Workshop Agenda**

[Facilitator Name Here]

**[Site Name Here]**

[Month Date], [Year]

[Start Time]-[End Time] **(2 Hours, B)**

**Agenda:**

9:00 - 9:10 Introductions

9:10 - 9:15 Group Agreements

9:15 - 9:20 Framing the Conversation

9:20 - 9:35 General Feelings About Pregnancy Options or The Last Abortion debrief

9:35 - 10:20 Abortion Patient Cases

10:20 - 10:50 Personal Challenges Discussion

10:50 - 11:00 Wrap UP

***Homework:*** *Review Group Agreements, relevant readings, General Feelings About Pregnancy Options or The Last Abortion*
