# Supplementary material for: Professionalism in Family Planning Care Workshop
Source: MedEdPORTAL. 2022 Jan 12;18:11212. doi: 10.15766/mep_2374-8265.11212 (PMC8752579; doi:10.15766/mep_2374-8265.11212)
Supplement: Supplementary file 1 — Editable Agendas.docxPFPCW Guide.docxProfessionalism Learner Presurvey.docxProfessionalism Learner Postsurvey.docxProfessionalism Facilitator Postsurvey.docxPFPCW Facilitator Training Video.mp4 [file mep_2374-8265.11212-s001.zip › B. PFPCW Guide.docx]

**
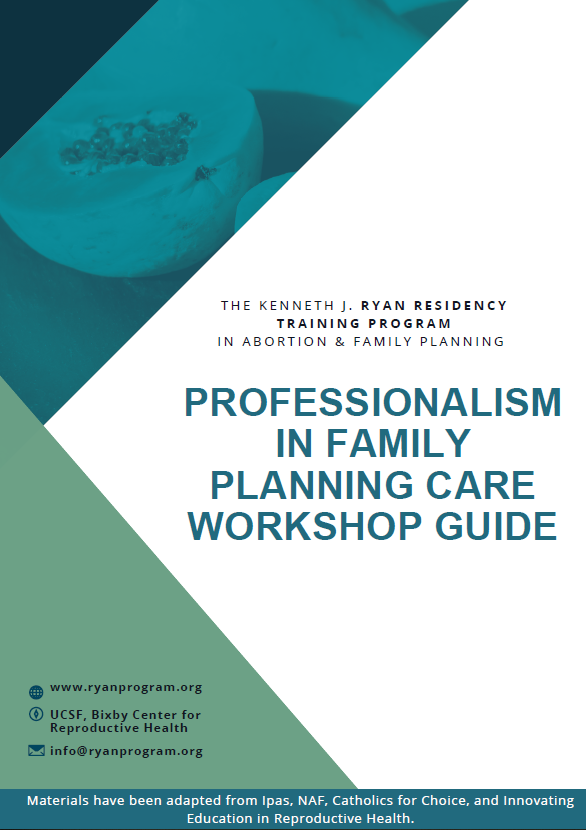
**Professionalism in Family Planning Care Workshop Guide

Contents

[Introduction 2](#_Toc80796823)

[Facilitation Guide 3](#_Toc80796824)

[Workshop Overview 4](#_Toc80796825)

[Sample Agendas 4](#_Toc80796826)

[Homework Options 6](#_Toc80796827)

[a) Readings 6](#_Toc80796828)

[b) Videos 6](#_Toc80796829)

[c) Interactive Values Clarification 7](#_Toc80796830)

[d) Exercise: The Last Abortion 7](#_Toc80796831)

[e) Exercise: Personal Reflection on Values 7](#_Toc80796832)

[f) Exercise: General Feelings about Pregnancy Options 7](#_Toc80796833)

[Workshop Components 7](#_Toc80796834)

[**Part 1: Introduction** 7](#_Toc80796835)

[**Part 2: Group Agreement** 7](#_Toc80796836)

[**Part 3: Framing the Conversation** 8](#_Toc80796837)

[**Part 4: Warm Up Activities** 9](#_Toc80796838)

[a) Hopes and Hesitations 9](#_Toc80796839)

[b) General Feelings about Pregnancy Options 10](#_Toc80796840)

[c) Cross the Line 11](#_Toc80796841)

[**Part 5: In-Depth Activities** 13](#_Toc80796842)

[a) Four Corners 13](#_Toc80796843)

[b) Abortion Patient Cases 15](#_Toc80796844)

[c) Challenging Cases 16](#_Toc80796845)

[d) The Last Abortion 17](#_Toc80796846)

[e) Comfort Continuum 17](#_Toc80796847)

[**Part 6: Personal Challenges** 20](#_Toc80796848)

[**Part 7: Wrap-Up** 21](#_Toc80796849)

[Additional Resources 22](#_Toc80796850)

[**I: Strategies for Managing Challenging Participants** 23](#_Toc80796851)

[**II: Physician Charter on Professionalism by the ABIM Foundation** 26](#_Toc80796852)

[**III: The Last Abortion: Worksheet** 27](#_Toc80796853)

[**IV: General Feelings about Pregnancy Options: Worksheet** 28](#_Toc80796854)

[**V: Four Corners: Worksheet** 29](#_Toc80796855)

# Introduction

We created the Professionalism in Family Planning Care Workshop (PFPCW) to support health professions learners to discuss challenging family planning cases so that they can provide patient-centered care when they feel negative emotions toward patients in future interactions. Healthcare providers’ negative feelings, such as frustration or anger, toward patients, can make it challenging to provide compassionate, high-quality care. In this workshop, learners discuss challenging patient interactions and family planning case scenarios to develop self-awareness and identify strategies for maintaining therapeutic relationships with patients when they find themselves experiencing these negative feelings. Upon completing the workshop, learners will be able to: (1) reflect on interactions in which they felt negative emotions toward patients, (2) reflect on their feelings about family planning clinical care scenarios, and (3) practice strategies such as understanding the patient context and finding empathy, for ensuring high-quality care for patients who make decisions about healthcare with which the provider may disagree.

We recommend framing the workshop around the concepts of professionalism and patient-centered care, that is to focus on their professional obligations to center the patient, even when they feel uncomfortable or frustrated with patients’ health decisions or behaviors. For each exercise in which a patient case is discussed we recommend asking the learners to articulate their reactions to or feelings toward the patient, as well as what they think is causing their feelings. This will help them develop self-awareness about reasons they might have negative emotions toward a patient. We then recommend exploring the patient context to find empathy for the patient. Why might the patient be making this decision or be in this circumstance? Depending on time available, this discussion may include exploring social and structural determinants of health, challenges in accessing healthcare, health literacy, and other important biopsychosocial issues. It also may include a discussion of clinical issues, for example, the side effects of contraceptives that might make a patient choose a specific method or no method. Finally, we recommend that facilitators emphasize their professional responsibilities in these scenarios to provide patient-centered care, protect patient autonomy, and put the patient at the center of the interaction. This can lead to a discussion of strategies they can use in these interactions such as shared decision making, that is, eliciting the patient’s values and supporting these values in their decision making.

The PFPCW can be modified by the facilitator as they wish. This facilitation guide includes many possible components from which the facilitator can choose. We include ideas for beginning and ending the workshop, homework options, warm-up activities, in-depth activities and a longer discussion activity. We recommend that every PFPCW begin with establishing group agreements for the workshop. Workshops typically last one to three hours, with one hour as the minimum suggested allotted time. When deciding on an agenda for the workshop, we recommend tailoring the activities based on the needs of the audience. This facilitation guide is intended to be used by facilitators as preparation and planning tools for their workshop, and to help walk facilitators through each workshop component. We also include facilitation skills. We hope this provides you with all the information needed to hold a successful PFPCW!

# Facilitation Guide

The following are tips for facilitating a Professionalism Workshop. We recommend that you review these tips and the following materials before leading a workshop.

- Facilitation requires that you guide learners through their own process, rather than instruct.
- Familiarize yourself with the materials and create a structure for the workshop in advance: preparation will help you feel more comfortable.
- Consider what type of learners or comments might be difficult for you and think about how you can interrogate your own reactions and respond in a calm, respectful manner.
- Group agreements are important to set the tone of the workshop, and referring back to them is a useful way to address disrespectful behavior.
- The schedule serves as a guide; don’t rush learners, but keep the conversation relevant to the workshop goals.
- Treat all learners with respect. Create an environment that is safe for learners to have open dialogue, question and reflect on their reactions/biases.
- Learners should be able to participate within their comfort level, but make sure to invite quieter learners to participate, and that a few learners do not dominate the conversation.
- Facilitation is often about adaptation; look for what the group is responding to and what is sparking good conversation. Address discomfort or concern that you sense in the group; this can be opened up to group problem solving.
- These workshops can be emotionally challenging. Learners may display strong emotions; allow them to leave the room if needed and acknowledge the difficulty of the workshop.
- This guide uses gender-neutral language such as “people”, “person” and the pronoun “they” to reflect the reality that people having abortions can be of any gender. Additionally, there are two scenarios involving non-binary and transgender-identifying folks. For the scenario with a transgender man, it can be useful to highlight that the scenario was written to reveal the difficulties of navigating the medical system while transgender. If learners are confused by the use of gender-neutral language, or any of the scenarios, you may use the above explanations.
- As a facilitator, you may also find this workshop to be emotionally challenging. Remember to take care of yourself and reach out to the Ryan Program for support if needed.
- As a facilitator, you are not expected to have all the answers. Invite learners to share their knowledge and take responsibility for addressing problems.

*(For Strategies for Managing Challenging Participants, see Appendix I)*

# Workshop Overview

This guide includes a variety of possible homework assignments, introductory exercises, warmup activities, in-depth exercises and a closing activity. We recommend that you select from the options to create your workshop. Exercises can also be assigned as homework or moved between warm-up and in-depth activities.

# Sample Agendas

Prior to the workshop the facilitator and the site will meet and tailor an agenda that fits best for the needs of that site and audience.

Some sample agendas are below.

1.5-hour Workshop

1. Opening Survey and Introduction from Ryan Program (5 minutes)
2. Introductions and Group Agreements (10 minutes)
3. Framing the Conversation (5 minutes)
4. Cross the Line (15 minutes)
5. Four Corners (30 minutes)
6. Personal Challenges Discussion (20 minutes)
7. Wrap up and closing survey (5 minutes)

2-hour Workshop

1. Opening survey and Introduction from Ryan Program (20 minutes)
2. Group Agreements (5 minutes)
3. Framing the Conversation (5 minutes)
4. General Feelings About Pregnancy Options (40 minutes)
5. Abortion Patient Cases (35 minutes)
6. Wrap up and closing survey (15 minutes)

# Homework Options

Select a few readings and videos that will be most relevant to your learners. Two optional exercises are included for reflection; if participants are asked to complete these, please include time during the workshop for a discussion of the exercise(s).

## Readings

- An Overview of Abortion Laws by Guttmacher Institute - <https://www.guttmacher.org/state-policy/explore/overview-abortion-laws>
- Harris, Lisa H, "Recognizing conscience in abortion provision," New England Journal of Medicine 367, no. 11 (2012): 981-983.

[http://www.nejm.org/doi/full/10.1056/NEJMp1206253#t=article](http://www.nejm.org/doi/full/10.1056/NEJMp1206253%23t=article%20)

## Videos

**Public Health Overview**

- Darney, Philip, “Preventing maternal mortality: The role of safe abortion,” Innovating Education in Reproductive Health

<http://innovating-education.org/2016/02/preventing-maternal-mortality-the-role-of-safe-abortion>

- DePiñeres, Teresa, “Abortion in the international context: Why, who and when?,” Innovating Education in Reproductive Health

<http://innovating-education.org/2016/02/abortion-in-the-international-context/>

- Joffe, Carole, “Abortion in the US after legalization,” Innovating Education in Reproductive Health

<http://innovating-education.org/2016/02/abortion-in-the-us-after-legalization/>

- Dehlendorf, Christine, “Abortion disparities: A public health framework,” Innovating Education in Reproductive Health

<http://innovating-education.org/2016/02/abortion-disparities-a-public-health-approach-2/>

- Drey, Eleanor and Dan Grossman, “Contextualizing who has abortions after the first trimester,” Innovating Education in Reproductive Health

<http://innovating-education.org/2016/02/contextualizing-who-has-abortions-after-the-first-trimester/>

**Decision Making and Education**

- The Framework: Counseling for Patient-Centered Abortion Care. Innovating Education in Reproductive Health. [https://www.innovating-education.org/2018/09/the-patient-has-the-answer-pregnancy-options-counseling-workshop/.](https://www.innovating-education.org/2018/09/the-patient-has-the-answer-pregnancy-options-counseling-workshop/.%20)
- Perrucci, Alissa, “Decision counseling for positive pregnancy test results,” Innovating Education in Reproductive Health

<http://innovating-education.org/2016/02/decision-counseling-for-positive-pregnancy-test-results/>

- Perrucci, Alissa, “Informed consent, decision assessment and counseling in abortion care,” Innovating Education in Reproductive Health

<http://innovating-education.org/2016/03/informed-consent-decision-assessment-and-counseling-in-abortion-care-2/>

**Professionalism**

- Steinauer, Jody, “Physicians’ professional responsibilities in abortion care,” Innovating Education in Reproductive Health <http://innovating-education.org/2016/02/physicians-professional-responsibilities-in-abortion-care/>

## Interactive Values Clarification

- Challenging Patient Encounters Digital Modules: Family Planning and Abortion [http://innovating-education.org/2016/04/values-clarification-web-module/](https://www.innovating-education.org/learning-module/values-clarification/)

## Exercise: The Last Abortion

This activity can be modified to be a homework option. Participants will reflect on the statements for homework, then discuss their responses in person *(see Appendix III for activity details).*

## Exercise: Personal Reflection on Values

- Where did your views on abortion come from? Which social group has had the greatest influence on your current values related to abortion? If there are differences between your feelings about abortion and the group that influenced you, how have they caused conflicts with members of these social groups?
- If you belonged to a spiritual/religious group while you were growing up, reflect on that group’s view on abortion. If you belong to a spiritual/religious group now, consider that group’s view on abortion.
- Consider your thoughts about parenthood and planning for parenthood. In what circumstances and at what age do you think a person should have a child? How many children do you think people should ideally have? How have your views about these topics changed over the years?

## Exercise: General Feelings about Pregnancy Options

This activity can be modified to be a homework option. Participants will reflect on the statements for homework, then discuss their responses in person *(see Appendix IV for activity details).*

# Workshop Components

**Part 1: Introduction**

Many different questions can be posed or strategies used for introductions. You can work with the site to choose an introductory exercise that is best tailored to your group.

**Part 2: Group Agreement**

Ask the group for an open discussion on Group Agreements and give them a chance to ask questions or make their own suggestions at the beginning of the workshop. If the following five points do not come up, include them.

- Only one person speaks at a time
- Step Up, Step Back: If you are someone who is often quiet in groups, challenge yourself to speak up. If you are someone who is very vocal in groups, be conscious about how much you are talking and give others the chance to speak first.
- Respect: Respect everyone, including yourself
- Confidentiality: What is said here, stays here
- Be Present: Turn off cellphones and devices and put them away
- For Virtual Workshops: Keep video on

**Part 3: Framing the Conversation**

This is a chance to provide a framework for the activities and discussion that will follow. Some ideas for framing are listed here:

- *We are all humans and therefore have opinions, beliefs and values about making decisions, living, parenting, healthcare, abortion, etc. We need to provide empathetic, patient-centered care to all patients, even when their decisions or behaviors make us uncomfortable. In order to do that it is helpful to take time to reflect on the things that upset us.*
- *As healthcare providers we have a number of obligations to patients. We must promote health, do no harm, and protect patient autonomy. It is our obligation to support patients to make decisions that are right for them, provide them information and support, provide healthcare services they need, and refer if we are unable to do so. We must also consider the ways in which we advocate for our patients to promote social justice and these ethical principles. When patient autonomy conflicts with our own beliefs we need to carefully reflect on this conflict and consider how we can promote patient autonomy and do no harm, while at the same time respect our personal beliefs. This is an opportunity to begin that exploration.*

This is a good time to have a brief discussion of their homework readings, potentially reviewing some basic information about abortion such as the following: *abortion is common, people have many reasons for having abortions, there are many restrictions that make it hard to access abortion, and access to safe abortion saves lives.*

## **Part 4: Warm Up Activities**

1. Hopes and Hesitations

Timeline

- **5 min.** to write on cards
- **5 min.** to talk in pairs
- **10 min.** to discuss responses

**Instructions**

1. On a flipchart, write the following:

- My overall hope for this workshop is…
- Right now, I feel hesitant about…
- I am concerned about being asked…
- I feel uncomfortable discussing…
- During the workshop, I hope that I will be able to…
- At the end of the workshop, I hope that I…

1. On another flipchart, write the headings ‘**Hopes**’ and **‘Hesitations’** in separate columns.

| HOPES | HESITATIONS |
| --- | --- |
|  |  |

Ask participants to share one hope or hesitation with the large group:

- - Record these on the flipchart labeled Hopes and Hesitations as each person speaks.
  - Write the responses exactly as they are stated.
  - *Remind participants that they may decline to share a response if they do not feel comfortable.*
- *Remind participants to refrain from commenting on or evaluating anyone’s response.*

1. After everyone who wants to has contributed, add your hopes for the workshop that were not mentioned by participants.
2. Ask for one or two overall comments about the entire list of hopes and hesitations (not any one person’s response).
3. Acknowledge that you will do your best to meet the group’s expectations.
   - Generally explain which agenda items should meet certain expectations and which may be beyond the scope of the workshop.
   - Record the latter items on the Parking Lot flipchart, if appropriate.
   - Reassure participants that you will discuss how they might meet these expectations in other ways outside of the workshop.
4. Let participants know that they should keep their index cards because they will refer to them at the end of the workshop as a means of checking if the workshop has helped to address their hopes and hesitations.
5. Solicit and discuss any outstanding questions, comments or concerns with the participants.
6. Thank the group for their participation.
7. General Feelings about Pregnancy Options

**Instructions**

*(See Appendix IV for activity details)*

1. Hand out worksheet to participants and have them mark the statements that are true for them.

- *Remind them they will not need to hand these in.*

1. Facilitate a discussion using some of the following questions:
   - What reasons for parenting make you uncomfortable, and what is the source of your discomfort?
   - What reasons for adoption make you uncomfortable, and what is the source of your discomfort?
   - What reasons for abortion make you uncomfortable, and what is the source of your discomfort?
   - How does this discomfort affect societal stigma against those who have abortions and providers who perform abortions?
   - How do you feel about a patient making a decision about their unintended pregnancy that they really don’t want to make?
   - (For participants working in reproductive health and abortion care) How does our discomfort with certain reasons affect our work in reproductive health and, specifically, abortion care? How might patients sense this discomfort? What impact could this have on the quality of healthcare we provide?
   - Was anyone surprised by their reactions to this question?
   - Were there any discrepancies? Did you find yourself more comfortable in the setting of a birth control failure than others? What about interference with education?
2. It’s okay to challenge learners. If a learner says she thinks it’s selfish to have an abortion just because it interferes with education, I ask: “Okay, what about if it interferes with their ability to finish high school? College? Medical School?”
3. If there are interesting discrepancies between pregnancy options, discuss.
4. Solicit and discuss any outstanding questions, comments or concerns with the participants.
5. Thank the group for their participation.
6. Cross the Line

*This activity is often used as an icebreaker to bring participants’ different views on abortion to the surface and address the connection between abortion and stigma. It helps participants understand how stigma affects people’s diverse views and experience with abortion, as well as broader public dialogue on abortion.*

**Objectives**

By the end of this activity, participants will be able to:

- Articulate their feelings and views on abortion;
- Identify diverse views among participants;
- Describe how stigma affects individual and societal views and reactions to abortion.

**Materials and Preparation**

- Masking tape or string, approximately 2-3 meters long, to mark a line on the floor. If neither tape nor string is available, ask participants to pretend that there is an imaginary line across the floor.
- Clear a large area of the room to allow participants to move around and place the line in the middle of this area.
- Review and adapt statements, if needed. Select in advance the statements you will read that most apply to that group of participants. It is advisable to end with a statement upon which you think all participants can agree, such as the last one in the list of statements below.
- For the virtual format have participants use the raise hand feature on Zoom.

**Instructions**

1. Ask all participants to stand on one side of the line.
2. Explain that you will read a series of statements and that participants should step entirely across the line when a statement applies to their beliefs or experiences.
3. Remind participants that there is no “in between,” which means they must stand on one side of the line or the other, and there are no right or wrong answers.
4. Ask participants not to talk during the exercise unless they need clarification or do not understand the statement that is read.
5. Stand at one end of the line and give an easy practice statement, such as: Cross the line if you had fruit for breakfast this morning.
6. Once some people have crossed the line, give participants an opportunity to observe who crossed the line and who did not.
7. Invite participants to notice how it feels to be where they are. Ask someone who crossed the line and then someone who did not to briefly explain their response to the statement. If someone is the only person who did or did not cross the line, ask them what that feels like.
8. Invite participants to all move back to one side of the line.
9. Repeat this for several of the statements about abortion. Select the statements that most apply to that group of participants.
10. After the statements are read, ask participants to take their seats.
11. Discuss the experience. Some discussion questions may include:

- How did you feel about the activity?
- What did you learn about your own and others’ views on abortion?
- Were there times when you felt tempted to move with the majority of the group?
- Did you move or not? How did that feel?
- What did you learn from this activity?
- What does this activity teach us about the stigma surrounding abortion?
- How might stigma affect people’s emotional experience with abortion? How would it affect their family members?
- How might stigma impact the experience of health workers and providers working in abortion care?

1. Debrief overall and on the last statement in particular. If everyone in the group crossed the line, discuss this commonality. If everyone did not cross the line, discuss how these different views affect people’s work on abortion care and the broader social climate for abortion in that setting.
2. Solicit and discuss any outstanding questions, comments or concerns with the participants.
3. Thank the group for their participation.

**Cross the Line Statements**

**Instructions**

Read some of the following statements, beginning each time with, “Cross the line if … ” After participants have moved, follow up each statement with, “observe who crossed the line and who did not … notice how it feels to be wherever you are … now please all move back to the same side of the line.”

**Cross the line if:**

- You were raised to believe that abortion should not be openly discussed
- At some point in your life, you believed abortion is wrong
- You were raised to believe that abortion is a human right
- You have been asked to keep someone’s abortion a secret
- You have ever felt uncomfortable talking about abortion
- You have ever felt embarrassed talking about abortion
- You have ever heard a politician talking in a derogatory manner about people who have had abortions
- You have ever heard a friend or family member talk in a derogatory manner about people who have had abortions
- You or someone you are close to has had an abortion
- You have ever stifled your feelings about an abortion experience
- You have ever avoided the topic of abortion to avoid conflict
- You have heard the term “baby killers” applied to people who have abortions or health workers who perform abortions
- At some point in your life, you believed that relief is a common reaction after abortion
- You believe there is a need for a supportive social environment for abortion
- You believe all people deserve access to safe, high-quality abortion services

***Activity adapted from:***

*Turner, Katherine L. and Kimberly Chapman Page. 2008. Abortion attitude transformation: A values clarification toolkit for global audiences. Chapel Hill, NC, Ipas.*

*Exhale. 2005. Teaching support: A guide for training staff in after-abortion emotional support. Oakland, CA, Exhale.*

## **Part 5: In-Depth Activities**

1. Four Corners

**Instructions**

1. Hand each participant a Four Corners worksheet. Ask them to complete the worksheet, crumple it into a ball, and throw it into the middle of the circle.
   - Randomly toss a “ball” back to each participant.
   - Explain that for the remainder of the activity, they will represent the responses on the worksheet they have in their hands.
   - If they got their own worksheet, they should act as though someone else completed it.
2. Read each statement out loud and ask participants to move to the sign that corresponds to the response circled on the worksheet they are holding.
   - You can hang the signs in each of the four corners of the room or you can place them on a continuum – Strongly Agree, then Agree, then Disagree, then Strongly Disagree.
3. Invite participants to look around the room and note the opinions held by the group.
   - There may be different-sized groups in each location, and sometimes all will not be occupied.
   - You can then ask some people to move to another group if the four are not evenly distributed.
4. For 2-3 statements, ask the group under each sign to discuss for two minutes the strongest rationale for why people might hold that opinion.
   - The Strongly Agree and Strongly Disagree groups should make sure they are brainstorming stronger opinions than their respective Agree or Disagree groups.
   - Ask each group to appoint a spokesperson to present why people might hold that opinion. Ask the spokespeople to speak convincingly, as though they hold the belief themselves. For example. “I strongly disagree with this statement because ...”
   - Ask each person to represent the thought respectfully, remembering that others in the room hold this opinion.
   - Start with the spokesperson under Strongly Agree and proceed in order to Strongly Disagree.
     - Remind participants that the designated spokespeople may or may not personally agree with the opinions they are presenting.
     - Do not allow other groups to comment at this time.
     - For each statement, reverse the order of the groups’ presentations.
5. Have participants return to their seats. Discuss the activity by asking some of the following questions:
   - What was it like to represent beliefs about abortion that were different from your own?
   - What was it like to hear your beliefs interpreted by others?
   - What rationale(s) for certain beliefs caused you to think differently?
   - What relevance do the beliefs discussed in this activity have for abortion care in our clinical setting? In our community? In our country?
   - (For healthcare providers and workers) How might our beliefs about abortion affect our provision of abortion-related services?
   - (For healthcare providers and workers) What can we do to ensure that we maintain a professional standard of high-quality reproductive healthcare regardless of our personal beliefs?
6. Solicit and discuss any outstanding questions, comments or concerns with the participants.
7. Thank the group for their participation.

*(See Appendix V for activity details)*

You can use our worksheet or modify it for your setting. Participants should mark Strongly Agree, Agree, Disagree, or Strongly Disagree for each statement.

These are some types of statements you can include: feelings or opinions about abortion in general.

Examples:

- People who have an abortion are ending a life.
- People who have an abortion after x weeks of gestation / who have more than one abortion are irresponsible.
- People who have multiple abortions should be encouraged to use birth control

Decision making autonomy

Examples:

- A person should be able to have an abortion even if their partner wants them to continue their pregnancy.
- Minors should be required to get their parents’ consent in order to have an abortion.

Professional requirements

Examples:

- Healthcare providers and workers who specialize in _______ (ob-gyn, family medicine, midwifery, adult medicine, reproductive health, use relevant specialty) have a responsibility to perform/participate in abortions. (You can also specify an abortion method).
- All medical/nursing/APC students should learn about abortion.

Policy

Examples:

- Third-trimester abortions should be legal and available.
- Abortion should be restricted after x weeks of gestation.
- People should have to wait 24 hours after counseling before they can get an abortion.

Training preparation and intentions

Examples:

- When I finish my training program I will be competent to provide first-trimester medication abortion
- When I finish my training program I will provide second-trimester abortion care

1. Abortion Patient Cases

**Cases**

- A 24-year-old person who has been pregnant three times and has had three abortions comes to you for pregnancy options counseling and desires an abortion.
- You are counseling a patient who desires an abortion. After you explain what to expect during the abortion you move on to discuss contraception. She tells you that she is not planning to use any contraception.
- You are a medical student rotating at an abortion clinic and a 23-year-old person comes in for termination of pregnancy at these gestational durations. Consider if you feel differently about each of them.
  - Gestational age is 8w1d
  - Gestational age is 23w0d (the last day for a termination at your clinic)

**Instructions**

1. Begin by asking participants:
   - What are your reactions?
   - How does the scenario make you feel?
   - How might your reactions and/or feelings come across to the patient?
2. For each scenario we discuss strategies for dealing with our reactions. Three strategies are useful to managing these reactions: Empathy, Compassion and Acceptance.
   - **Empathy:** Try to put yourself in the patient’s shoes.
   - **Compassion:** If you can’t put yourself in her shoes, can you at least understand that she is having a hard time or just needs your support?
   - **Acceptance:** If these fail, can you simply accept her behavior? If all of these fail, you may need to refer care. What is a way to do that professionally?
3. You can encourage discussion with the following questions/prompts:
   - Let’s explore the patient’s perspective. What do you think is going on for this patient? Let’s brainstorm.
   - What strategies can healthcare providers use to maintain a healthy relationship with the patient? What might you do the next time you interact with a similar patient?
   - What can you do to recover from a judgmental moment with a patient?

As before, it is important to emphasize the normalcy of reacting and feeling judgmental.

This could be an opportunity to reflect on The Last Abortion exercise the learners did for homework (15min)

1. Challenging Cases

**Instructions**

1. Pose the following questions to the group:
   - Tell us about an experience in which you felt frustrated with or angry with a patient. How did you handle it? How might this have affected your care of this patient?
   - Tell us about a time when you observed a resident or attending react toward a patient in a way that negatively affected their relationship with the patient.
2. As each learner presents their case it is important to emphasize the normalcy of reacting and feeling judgmental; even exclaim things like, “Wow, that’s quite a reaction! Great!”

Frequently discussed scenarios include:

- - Patients who don’t always tell us the truth or the full story
  - Parents who they feel are putting their children at risk (for example, choosing to not vaccinate)
  - People who have had more than one abortion or choose to not use birth control or to use a method different from one the provider thinks they should use
  - Patients who don’t do what healthcare providers think is best (for example, surgery for endometrial cancer)
  - People who use substances while pregnant
  - Patients who continue to use substances despite education
  - Medication non-adherence

1. For each scenario, we discuss strategies for dealing with our reactions:
   - What are your reactions? How does the scenario make you feel? [mindfulness/self-awareness]
   - How might your reactions and/or feelings come across to the patient?
   - Let’s explore the patient’s perspective. What kinds of questions could you ask the patient? What might be going on with the patient that would explain her behavior? [empathy/compassion]
   - What strategies can you come up with to maintain a healthy relationship with the patient? What could you do next time you interact with a similar patient?
   - What can you do when you feel that your reactions might have harmed the relationship?
2. You are welcome to include your own teaching points about whatever misconceptions they may have but try to focus on their reactions.

This exercise allows you to remind the group that their reactions can get in the way or harm a patient-provider relationship and that they should not interpret these modeled behaviors as ideal behaviors. It also introduces the connection to professionalism. *These prompts can also be used for a homework activity, with a 10-15 minute debrief in person.*

### The Last Abortion

*(See Appendix III for activity details)*

**Instructions**

1. Have students share their vote by a show of hands. Tally up which individuals were given an abortion on a board or flipchart so the larger group can see the results.
2. Participants will likely feel frustrated that they could choose only one patient, however you can highlight how having to choose forces reflections on priorities and judgements that inform what determines a “good”/’better”/”worse”/”bad” or “deserving”/”more or less deserving” abortion.
3. Have one student explain why they chose as they did for each patient.
4. Facilitate a discussion using the following questions:
   - What made this exercise hard?
   - What factors played a role in deciding who should ultimately have the abortion?
   - Would it be different if you had to pick one to be a parent? Or one to choose placing the baby for adoption?

### Comfort Continuum

*This activity is designed to help participants reflect on their level of comfort discussing, advocating for and/or providing abortion services. Participants are encouraged to reflect on their life experiences that influenced these comfort levels and how they relate to societal norms on abortion.*

**Objectives**

By the end of this activity, participants will be able to:

- Articulate their own comfort levels discussing or advocating for safe abortion services;
- Discuss the different comfort levels on abortion held by participants and the life experiences that inform them;
- Discuss how these varying comfort levels relate to societal norms on abortion;
- For healthcare providers - express their personal levels of comfort providing abortion care.

**Timeline**

- 20 minutes to complete the group activity.
- 20 minutes to discuss the activity.

**Materials**

- Three paper signs labeled “Not At All”, “A Little”, “A Lot”.
- Tape
- Comfort Continuum statements

**Advance Preparation**

- Label three signs on paper: “Not At All”, “A Little”, “A Lot”.
- Rearrange chairs and tables, if necessary, to create an open space in the room for participants to move around.
- Review and revise statements, if necessary, selecting statements that are most relevant for this group of participants and the specific topics covered in your workshop. (There are two sets of statements, one that is more appropriate for healthcare providers and workers, and the other for reproductive health professionals or a general audience.)
- Prepare the statements you will read and the order in which you will read them.
- You may want to read only five to eight statements, as too many may make the exercise less interesting.
- Begin with easier statements, and then progress to harder or more complicated ones. It is advisable to use an overarching, final statement, such as the one listed here.
- Prepare correct information on abortion laws and policies in the country in case questions arise.

_______________________________________________________________________________________

*Note to facilitators: You may have to change or reword some of the statements in order to fit the*

*context of the country or community you are working in.*

________________________________________________________________________________________

**Instructions**

1. Tape the three signs on the floor or the wall in an open area of the room where there is enough room for participants to move around. Place the signs in order in a row to indicate a continuum:


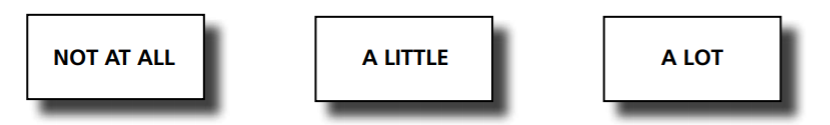


1. One at a time, read aloud the statements and ask participants to physically move to the point along the continuum that best represents their feelings. Encourage participants to be honest about their feelings and to resist being influenced by where other participants are placing themselves.
2. After participants have arranged themselves, ask volunteers at different points along the continuum to explain why they are standing there.
3. If, based on someone’s explanation, participants want to move to another point on the continuum, encourage them to do so.
4. Once you have finished reading the statements, ask participants to return to their seats. Ask two participants to share their feelings about the activity, soliciting a different response from the second person.
5. Refer to the reasons participants gave about their place on the continuum as you facilitate a brief discussion about the different responses and levels of comfort in the room. Some discussion questions could include:

- What observations do you have about your own responses to the statements? Other people’s responses?
- Were there times when you felt tempted to move with the majority of the group? Did you move or not? How did that feel?
- What about your responses to the statements surprised you? How about other people’s responses?
- What did you learn about your own and others’ comfort levels on abortion?
- What observations do you have about the group’s overall level of comfort with abortion (not individual people’s responses)?

1. Ask participants to reflect on the life experiences that influenced their levels of comfort or discomfort. Invite them to imagine how a different set of life circumstances might have led to a different level of comfort with abortion. Ask a few people to share their thoughts on this.
2. Discuss how these different levels of comfort with abortion impact societal norms on abortion, people’s feelings about themselves when they have an abortion and providers’ feelings about performing abortion services.
3. If participants are healthcare providers, facilitate a discussion on how their comfort levels impact the provision and quality of abortion services. Emphasize what a large impact providers’ attitudes have on their provision of services and patient’s experience and satisfaction with those services.
4. If questions arise during the discussion, for example on abortion laws and policies in that country, be prepared to provide correct information once participants have finished the discussion.
5. Ask one or two participants to share what they learned from this activity.
6. Solicit and discuss any outstanding questions, comments or concerns with the participants.
7. Thank the group for their participation.

**Comfort Continuum: Statements for Healthcare Providers and Health Workers**

**Facilitator** **Instructions**

Below are statements appropriate for healthcare providers and health workers. You can choose some of the following statements or develop other statements that are most relevant in your country or setting.

1. How comfortable are you with safe and legal induced abortion services being provided in your country?
2. How comfortable are you discussing abortion with colleagues at work?
3. How comfortable are you discussing abortion outside of your work setting?
4. How knowledgeable are you about your country’s laws and policies on abortion services?
5. How comfortable are you working in a facility where abortions are performed?
6. How much disapproval would you expect to feel from your family and friends if you provided (or assisted with) abortion services?
7. How comfortable are you performing an abortion in the first trimester? If you are not authorized to perform first-trimester abortion in your country, how comfortable are you assisting with a first-trimester abortion?
8. How comfortable are you performing an abortion in the second trimester? If you are not authorized to perform second-trimester abortion in your country, how comfortable are you assisting with a second-trimester abortion?
9. How comfortable are you with the idea of every person having the right to access safe abortion services in your country?
10. How comfortable are you providing (or assisting with) abortion for every person who desires it, regardless of her reasons?

**Comfort Continuum: Statements for Reproductive Health Professionals or General Audience**

**Facilitator Instructions**

Below are statements appropriate for reproductive health professionals or a general audience. You can choose some of the following statements or develop other statements that are most relevant in your country or setting.

1. How comfortable are you with safe and legal induced abortion services being provided in your country?
2. How comfortable are you discussing abortion with family members?
3. How comfortable are you discussing abortion with work colleagues?
4. How knowledgeable are you about your country’s laws and policies on abortion services?
5. How comfortable are you advocating for access to first-trimester abortion?
6. How comfortable are you advocating for access to second-trimester abortion?
7. How comfortable are you publicly supporting people who have abortions and the healthcare providers who provide them?
8. How much disapproval would you expect to feel from your family and friends if you advocated for safe abortion services?
9. How comfortable are you with the idea of everyone having the right to access safe abortion services in your country?
10. How comfortable are you advocating for abortion care for every person who desires it, regardless of her reasons?

***Activity adapted from:***

*Turner, Katherine L. and Kimberly Chapman Page. 2008. Abortion attitude transformation: A values clarification toolkit for global audiences. Chapel Hill, NC, Ipas.*

*Marais, Thea. 1996. Abortion values clarification training manual. Melrose, South Africa, Planned*

*Parenthood Association of South Africa*

## **Part 6: Personal Challenges**

If it is appropriate for the group, they might want to have a discussion of their feelings about abortion or any other cases where they have had personal challenges in their decisions about providing abortions. This is a great activity to highlight the way judgements and bias come up for all patients, not just abortion cases, and broaden the scope to allow participants to think about how their personal attitudes can affect patients beyond abortion care.

## **Part 7: Wrap-Up**

*This is a time for addressing questions, having a closing discussion, and giving participants a chance to process the workshop.*

Solicit input from the group on the following questions:

- What is one new perspective or thought that you can take away from the workshop?
- What came up for you that was surprising?
- What are you left wondering?
- What are your plans for the future care of patients that might now be different?
- What are you thinking about how care is provided in your setting?
- Do you want to keep meeting on some kind of regular basis to discuss these issues?

Have the group complete the post-workshop survey.

# **Additional Resources**

## **I: Strategies for Managing Challenging Participants**

**Strategies for Managing Challenging Participants**

**Table from Turner, Katherine L. and Kimberly Chapman Page. 2008. Abortion attitude transformation: A values clarification toolkit for global audiences. Chapel Hill, NC, Ipas.*

| **Types of challenging participants** | **Why are they challenging?** | **Strategies to effectively manage this type of participant** |
| --- | --- | --- |
| **“Know-it-alls”** | • May actually have a lot of  information about the topic,  but still could benefit from  the experiences and  perspectives of others | • Acknowledge that they know a wealth of information  • Approach them during a break and ask for their assistance in answering a  specific question   - At the same time, express that you want to encourage everyone to participate and enlist their help in doing so |
| **“I’m only here**  **because I have to**  **be”** | • May have been required to  attend the workshop, yet  have no particular interest in  the topic | • Acknowledge that you know that some of the participants are present because they have to be  • Ask for their assistance in making this a meaningful experience  • Ask specifically, ‘How can I make this workshop helpful to you?’ |
| **“Naysayers”** | • May be prejudiced  • Won’t accept your or other  participants’ points of view  • Unwilling to negotiate or compromise on their  position  • Often disruptive and create  discomfort for the group | • Do not put them down or make them feel isolated   - Keep them involved, if possible   • Throw their views to the group by questions or examples   - Try to get the group to bring them around   • Say that time is short and you would be glad to discuss their issues with them  individually  • Ask them to accept the views of the group or the trainer for the moment |
| **“Talkers”** | • May be ‘eager beavers’ or  show-offs  • May be exceptionally well-informed and anxious to  show it or just naturally  wordy  • May need to be heard  because they are still  working through difficult  emotional issues  • May take time away from  other participants | • Do not be belittling or sarcastic as you may need their help later  • Slow them down with some difficult question or task, such as group leader  • Interrupt tactfully with a comment like, ‘That’s an interesting point...now let’s see what the rest of the group thinks of it”  • In general, let the group take care of them as much as possible  • Avoid eye contact  • Give them a role  • State that one of your roles is to keep people on time  • Quick interruption (i.e. move nearby and put your hand on his or her shoulder)  • Paraphrase what they say and move on  • Acknowledge that their stories are important, and you and others would love to hear them later or after the workshop |
| **“Inaccurate**  **commentators”** | • Come up with comments  that are obviously incorrect | • Say, ‘Thank you for giving me a chance to clear up that point’  • Say, ‘I see your point, but can we look at it this way…’  • Don’t ever put them down or make them feel stupid   - They must be handled positively and delicately   • Ask if others have the same belief  • Acknowledge what they have offered as a common myth or commonly misunderstood concept |
| **“Clashers”** | • Two or more participants  strongly disagree or bring  personal conflicts into the  discussion. This can divide  your group into factions | • Emphasize points of agreement; minimize points of disagreement  • Point out how the argument has been productive in illustrating certain points  • Draw attention to session objectives and group norms; cut across the argument with a direct question about the topic  • Bring a less argumentative participant into the discussion  • Remain calm   - Ask participants to refrain from personal attacks and to keep arguments productive and directed toward topic definition or resolution   • Stay neutral  • Stick to the topic  • Acknowledge emotionality of topic |
| **“Side**  **conversationalists”** | • Have conversations with  their neighbors that may or  may not be related to the  topic, but are distracting to  other participants or to you | • Do not embarrass them  • Call them by name; ask an easy question  • Call them by name, then restate the last opinion expressed or last remark made by group, and ask their opinion of it  • If you are in the habit of moving around the room, saunter over and stand casually behind them   - This should make their conversation obvious to them and the group   • Ask the group to add “no side conversations” to the list of ground rules |
| **“Questioners”** | • May be genuinely curious  • May be testing you by  putting you on the spot  • May have an opinion, but  lack the confidence to  express it | • Acknowledge that they seem to have a lot of questions about a particular topic  • If the questions seem like legitimate attempts to gain content information that other members of the group already know, tell them that you will be happy to work with them later to fill in the gaps, or put the question on the parking lot flip chart to be discussed at a later time  • Reframe or refocus by sending the questions back to the questioner  • Establish a buddy system (for example, ask for volunteers who would be willing to meet with them) |
| **“Ramblers”** | • Talk about everything but the topic  • Use inappropriate or  farfetched examples from  their own experience | • When they stop for a breath, thank them, then refocus attention by restating  relevant points and move on  • Smile; tell them that their points are interesting, apply them to the discussion, if you can, and indicate in a friendly manner that the group is getting a bit off subject |
| **“Shy and timids”** | • May feel timid or insecure  • May be bored or indifferent | • Try to arouse their interest by asking them an easy, direct question   - Talk to them on a personal basis with the group looking on   • Ask questions of the person next to them, and then ask them to respond to  that person’s answer |
| **“Off-base**  **commentators”** | • Are not rambling, but make  comments that are not  relevant to discussion  • May confuse other  participants | • Say, ‘How would you relate this to the discussion at hand?’  • Say, ‘It sounds like what you are saying is…’ and then rephrase. Then clarify, ‘Is that a fair statement of your point?’  • Set aside the comment or question for later discussion  • Reframe or refocus onto the topic  • Explain where the participants’ comments fit into the curriculum |
| **“Arguers”** | • Have combative personalities  • May not want to be at the  workshop  • May be upset by personal or family health issues  • May upset other participants | •Keep your own temper firmly in check   - Do not let the group get excited either   • Honestly try to find merit in one of their points, or get the group to do it, and then move on to something else   - Say, ‘That was a good point’ or ‘We heard a lot from [person’s name]. Who else has some ideas?’   • If facts are misstated, ask the group for their thoughts   - Let the group make the corrections   • As a last resort, talk with them in private, find out what’s going on and ask for their cooperation   - Say, ‘Let’s talk during the break. How can we be on the same team?”   • Give them a role |
| **“Gripers”** | • Have a pet peeve with you,  the group, the subject, the  healthcare system, etc. | • Indicate that you’ll discuss the problem with them in private later  • Throw the issue back to the group and have a member of the group answer them  • Indicate time pressures and emphasize the need to move on |
| **“Emotionals”** | • May upset other participants | • Make sure they feel free to leave the room if they find it is necessary to take care of themselves  • Allow other participants to comfort them  • Encourage them to talk with you or others during breaks or at the end of the workshop |

## **II: Physician Charter on Professionalism by the ABIM Foundation**

We have listed the fundamental principles and the summary from the Physician Charter on Professionalism by the ABIM Foundation here. For the full text of the charter, please reference this link - https://abimfoundation.org/what-we-do/physician-charter

**Fundamental Principles**

**Principle of primacy of patient welfare.**

The principle is based on a dedication to serving the interest of the patient. Altruism contributes to the trust that is central to the physician-patient relationship. Market forces, societal pressures, and administrative exigencies must not compromise this principle.

**Principle of patient autonomy.**

Physicians must have respect for patient autonomy. Physicians must be honest with their patients and empower them to make informed decisions about their treatment. Patients’ decisions about their care must be paramount, as long as those decisions are in keeping with ethical practice and do not lead to demands for inappropriate care.

**Principle of social justice.**

The medical profession must promote justice in the healthcare system, including the fair distribution of healthcare resources. Physicians should work actively to eliminate discrimination in healthcare, whether based on race, gender, socioeconomic status, ethnicity, religion, or any other social category.

**Summary**

The practice of medicine in the modern era is beset with unprecedented challenges in virtually all cultures and societies. These challenges center on increasing disparities among the legitimate needs of patients, the available resources to meet those needs, the increasing dependence on market forces to transform healthcare systems, and the temptation for physicians to forsake their traditional commitment to the primacy of patients’ interests. To maintain the fidelity of medicine’s social contract during this turbulent time, we believe that physicians must reaffirm their active dedication to the principles of professionalism, which entails not only their personal commitment to the welfare of their patients but also collective efforts to improve the healthcare system for the welfare of society. This Charter on Medical Professionalism is intended to encourage. This Charter on Medical Professionalism is intended to encourage such dedication and to promote an action agenda for the profession of medicine that is universal in scope and purpose.

## **III: The Last Abortion: Worksheet**

Each of the following people has decided to have an abortion. You must choose which person will be able to receive the last safe, legal abortion. You can only choose one candidate (and you must choose). The importance of choosing is to interrogate the thought processes that led to that choice and the biases and judgements underlying what constitutes a “good or deserving” abortion. This can be unpacked throughout the exercise.

1. A 45-year-old woman is 18-weeks pregnant. She had stopped having regular menstrual cycles and did not believe she could become pregnant. An ultrasound shows that her fetus has severe fetal anomalies. The woman shares that she is not able to manage this pregnancy nor parent a child who will require complex medical care and likely have disabilities.
2. A 28-year-old woman is 12-weeks pregnant. She does not want to have children or be a parent. She has had two previous pregnancies and two previous abortions.
3. A 21-year-old non-binary person in their third year of university just found out that they are 14-weeks pregnant. Because their menstrual cycle was irregular, they did not realize they were pregnant. Their contraceptive method failed, even though they are quite certain they used it properly. They are experiencing acute anxiety at the thought of continuing this pregnancy.
4. A 25-year-old woman is 8-weeks pregnant. She lives with her partner and their two children. Her partner is abusive and does not want her to have an abortion. She does not want to have another child with this person nor be dependent on them for financial support.
5. A 35-year-old woman is 6-weeks pregnant. She has two children, a 15-year-old and an 11-year-old. She is working full time and has recently returned to school to get a master’s degree. She knows her family is complete and does not want any more children.
6. A 15-year-old girl is 14-weeks pregnant as a result of rape by her stepfather. When she told her mother about the rape and pregnancy, her mother told her to get out of the house. She has been staying at a friend’s house and continues to attend school
7. A 23-year-old transgender man is pregnant and concerned about the pregnancy interfering with his hormone therapy and transition. He is unable to find adequate resources and support for his questions and concerns from the medical community.
8. A 17-year-old had sex for the first time with their partner and got pregnant. They are now 15-weeks pregnant and are not ready to be a parent. They were raised believing that abortion was “murder” and feel conflicted about their desire to have an abortion.

## **IV: General Feelings about Pregnancy Options: Worksheet**

*Please mark the statements that are true for you:*

- **I am generally comfortable with a patient choosing to have an abortion in the following circumstances:**
  - If the pregnancy threatens her life.
  - If the pregnancy threatens her physical or mental health.
  - If the pregnancy involves fetal abnormalities.
  - If the pregnancy resulted from rape.
  - If she does not want any more children.
  - If she is financially unable to support a child.
  - If the baby would interfere with education or career goals.
  - If the pregnancy resulted from a birth control failure.
  - If she does not want a child of a particular sex.
- **I am generally comfortable with a patient giving the baby for adoption in the following circumstances:**
  - If the pregnancy threatens her life.
  - If the pregnancy threatens her physical or mental health.
  - If the pregnancy involves fetal abnormalities.
  - If the pregnancy resulted from rape.
  - If she does not want any more children.
  - If she is financially unable to support a child.
  - If the baby would interfere with education or career goals.
  - If the pregnancy resulted from a birth control failure.
  - If she does not want a child of a particular sex.
- **I am generally comfortable with a patient choosing parenthood in the following circumstances:**
  - If the pregnancy threatens her life.
  - If the pregnancy threatens her physical or mental health.
  - If the pregnancy involves fetal abnormalities.
  - If the pregnancy resulted from rape.
  - If she does not want any more children.
  - If she is financially unable to support a child.
  - If the baby would interfere with education or career goals.
  - If the pregnancy resulted from a birth control failure.
  - If she does not want a child of a particular sex.

## **V: Four Corners: Worksheet**

*Please take a few minutes to read the following statements and mark each one with* ***Strongly Agree, Agree, Disagree or Strongly Disagree.*** *This worksheet will be anon*ymously submitted; please do not write your name.

1. **A person who has an abortion is ending a life.**
   - Strongly Agree
   - Agree
   - Disagree
   - Strongly Disagree
2. **Healthcare providers and workers who specialize in ob-gyn have a responsibility to perform abortions.**
   - Strongly Agree
   - Agree
   - Disagree
   - Strongly Disagree
3. **Minors should be required to get their parents’ consent in order to have an abortion.**
   - Strongly Agree
   - Agree
   - Disagree
   - Strongly Disagree
4. **A person should be able to have a second-trimester abortion if they need one.**
   - Strongly Agree
   - Agree
   - Disagree
   - Strongly Disagree
5. **A person who has had more than one abortion should be encouraged to use birth control.**
   - Strongly Agree
   - Agree
   - Disagree
   - Strongly Disagree
6. **When I finish my training program, I will provide medication abortion care to patients.**
   - Strongly Agree
   - Agree
   - Disagree
   - Strongly Disagree
7. **When I finish my training program, I will be competent to provide second-trimester D&E abortion care.**
   - Strongly Agree
   - Agree
   - Disagree
   - Strongly Disagree
