# Supplementary material for: Professionalism in Family Planning Care Workshop
Source: MedEdPORTAL. 2022 Jan 12;18:11212. doi: 10.15766/mep_2374-8265.11212 (PMC8752579; doi:10.15766/mep_2374-8265.11212)
Supplement: Supplementary file 1 — Editable Agendas.docxPFPCW Guide.docxProfessionalism Learner Presurvey.docxProfessionalism Learner Postsurvey.docxProfessionalism Facilitator Postsurvey.docxPFPCW Facilitator Training Video.mp4 [file mep_2374-8265.11212-s001.zip › C. Professionalism Learner Presurvey.docx]

# Ryan Program Professionalism Workshop - Pretest

Please complete this pre-workshop survey.

This survey will assess your experiences before a Professionalism Workshop. The estimated average time to complete the survey is 10 minutes. The data resulting from this evaluation allows us to improve the overall quality of Professionalism Workshops. All information we receive will be handled confidentially. Completed surveys are kept in a password protected database. Any publications or presentations resulting from this study use only aggregate data. No institutions or individuals will be identified.

Your return of a completed survey will indicate your consent to participate in the study. 2020

##

1. Please enter your institution name:

# Ryan Program Professionalism Workshop - Pretest

3. For survey matching purposes only, please provide the last four digits of your cell phone number. *Your survey responses will be de-identified, thus will remain anonymous and confidential.

Last 4 digits of cell phone


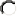

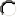

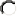

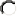

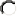

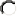

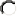

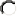

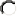

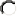

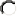

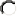

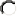

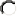

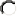

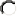


4. Which of the following best describes your current role?

Medical student Ob-gyn resident

Family medicine resident Resident in other specialty Undergraduate nursing student Physician assistant student Nurse midwifery student Nurse practitioner student Fellow (family planning)

Fellow (non-family planning) Physician

Nurse Midwife

Nurse practitioner Physician assistant Other

# Ryan Program Professionalism Workshop - Pretest


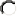

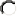

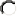

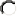

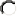

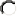

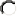


5. If you are currently in a training program, which year are you currently?

1st 2nd 3rd 4th 5th 6th

Not in training program

# Ryan Program Professionalism Workshop - Pretest

6. Please list your specialty and your subspecialty, if relevant.


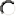

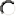


7. Have you attended a workshop previously that discussed abortion or challenging patients, such as a Values Clarification, before? If yes, when and how many times?

No

Yes

If yes, when and how many times?

**Below you will find 4 scenarios^[[1]](#footnote-1)^ describing different types of patient behaviors. Please read the patient scenario and then indicate your level of agreement with the statements below.**


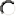

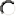

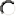

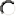

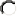

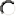

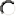

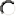

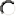

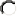

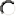

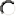

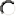

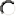

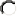


8. A patient who has an undesired pregnancy and chooses to have an abortion.

Strongly disagree

Neutral

Strongly agree

I can think of justifiable reasons that would explain why the patient is in this circumstance and makes this decision.

This case makes me feel frustrated.

My reaction to this case would make it hard for me to care for this patient.


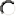

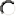

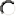

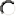

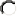

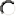

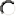

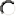

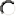

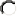

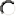

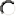

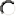

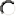

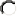


9. A patient who just underwent an abortion and is not interested in starting a birth control method.

Strongly disagree

Neutral

Strongly agree

I can think of justifiable reasons that would explain why the patient is in this circumstance and makes this decision.

This case makes me feel frustrated.

My reaction to this case would make it hard for me to care for this patient.


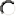

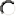

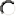

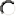

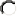

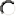

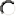

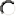

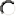

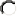

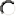

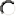

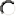

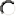

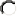


10. A patient who has had an abortion in the past and now presents with an undesired pregnancy and wants an abortion.

Strongly disagree

Neutral

Strongly agree

I can think of justifiable reasons that would explain why the patient is in this circumstance and makes this decision.

This case makes me feel frustrated.

My reaction to this case would make it hard for me to care for this patient.


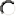

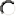

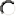

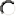

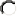

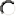

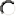

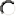

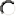

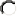

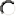

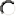

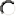

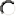

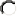


11. A patient who has an undesired pregnancy and presents for care for the first time at 19 weeks’ gestation.

Strongly disagree

Neutral

Strongly agree

I can think of justifiable reasons that would explain why the patient is in this circumstance and makes this decision.

This case makes me feel frustrated.

My reaction to this case would make it hard for me to care for this patient.


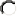

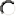

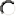

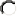

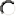

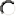

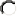

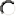

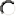

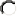

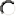

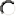

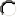

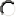

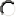


12. The following section contains questions regarding your participation in abortion-related care in specific procedures and situations in the past, as well as intentions to participate and provide services in the future.

"Medically indicated" refers to cases for maternal health, fetal anatomic, and genetic anomaly reasons.

In the past, have you directly provided:

Yes

No

I don't know

Comprehensive pregnancy options counseling

First-trimester medication abortion for non-medical reasons

First-trimester medication abortion for medically indicated cases

First-trimester uterine aspiration for non-medical reasons

First-trimester uterine aspiration for medically indicated cases

Second-trimester D&E for non-medical reasons

Second-trimester D&E for medically indicated cases

Second-trimester induction for non- medical reasons

Second-trimester induction for medically indicated cases

Referral for abortion care

13. Do you intend to directly provide the following procedures in the future -- in training and/or future practice (if your practice allows)?

"Medically indicated" refers to cases for maternal health, fetal anatomic, and genetic anomaly reasons.

[1] [2]

Certainly no Probably no

[3] [4] [5]

Neutral Probably yes Certainly yes

Comprehensive pregnancy options counseling

First-trimester medication abortion for non-medical reasons

First-trimester medication abortion for medically indicated cases

First-trimester uterine aspiration for non-medical reasons

First-trimester uterine aspiration for medically indicated cases

Second-trimester D&E for non-medical reasons

Second-trimester D&E for medically indicated cases

Second-trimester induction for non- medical reasons

Second-trimester induction for medically indicated cases

Referral for abortion care

Disclaimer:

The following question uses a previously validated scale of abortion attitude^[[2]](#footnote-2)^ and may not fully encompass your personal beliefs. Please answer these questions to the best of your ability.

14. The following is a list of reasons a patient may ask for an abortion. Using the given 5-point scale (1= strongly disagree, 5= strongly agree), please indicate whether you agree or disagree that the stated reason is morally acceptable.

[1] Strongly [2] Somewhat disagree disagree

[4] Somewhat [5] Strongly

[3] Neutral agree agree

A patient is financially unable to support the child.

The patient's career/education would be disrupted.

The patient already has too many children.

The pregnancy is a result of rape or incest.

The pregnancy is a threat to the patient's physical health.

15. How important is religion in your life?

Very unimportant

Unimportant

Neutral

Important

Very important

16. What is your religious affiliation?

Protestant Roman Catholic Mormon Orthodox Jewish

Muslim Buddhist Hindu Atheist Agnostic None

Prefer not to answer Other

17. How often do you attend church or other religious meetings?

Never

One a year or less A few times a year

A few times a month Once a week

More than once a week Prefer not to answer Other

## The following statements are part of a validated scale of empathy^[[3]](#footnote-3)^. For each item, please indicate how well it describes you. Answer as honestly as you can.

##

1. The following statements are part of a validated scale of physician's beliefs^[[4]](#footnote-4)^ in the psychosocial problems related to patient care. **Please read the following statements and indicate your level of agreement. Answer as honestly as you can.**

1. Case scenarios created by Dr. Jody Steinauer and the Ryan Residency Training Program National Office Staff [↑](#footnote-ref-1)
2. Adapted by authors from: Aiyer AN, Ruiz G, Steinman A, Ho GY. Influence of physician attitudes on willingness to perform abortion. *Obstetrics and gynecology.* 1999;93(4):576-580. [↑](#footnote-ref-2)
3. Adapted by authors from: Davis M. A Multidimensional Approach to Individual Differences in Empathy. *JSAS Catalog Sel Doc Psychol.* 1980;10. [↑](#footnote-ref-3)
4. Adapted by authors from: Ashworth CD, Williamson P, Montano D. A scale to measure physician beliefs about psychosocial aspects of patient care. *Soc Sci Med.* 1984;19(11):1235-1238. [↑](#footnote-ref-4)
