# Supplementary material for: Professionalism in Family Planning Care Workshop
Source: MedEdPORTAL. 2022 Jan 12;18:11212. doi: 10.15766/mep_2374-8265.11212 (PMC8752579; doi:10.15766/mep_2374-8265.11212)
Supplement: Supplementary file 1 — Editable Agendas.docxPFPCW Guide.docxProfessionalism Learner Presurvey.docxProfessionalism Learner Postsurvey.docxProfessionalism Facilitator Postsurvey.docxPFPCW Facilitator Training Video.mp4 [file mep_2374-8265.11212-s001.zip › D. Professionalism Learner Postsurvey.docx]

# Ryan Program Professionalism Workshop - Post Test

Please complete this post-workshop survey.

This survey will assess your experience during and after a Professionalism Workshop. The estimated average time to complete the survey is 5 minutes. The data resulting from this evaluation allows us to improve the overall quality of Professionalism Workshops. All information we receive will be handled confidentially. Completed surveys are kept in a password protected database. Any publications or presentations resulting from this study use only aggregate data. No institutions or individuals will be identified.

Your return of a completed survey will indicate your consent to participate in the study. 2020

1. Please enter the date of the workshop you attended (mm/dd/yy)

2. For survey matching purposes only, please provide the last four digits of your cell phone number. *Your survey responses will be de-identified, thus will remain anonymous and confidential.

Last 4 digits of cell phone

# Ryan Program Professionalism Workshop - Post Test

3. Please enter your institution name:


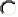

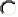

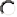

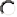

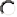


4. How useful was the workshop you attended, overall?

Extremely Useless

Somewhat Useless

Ambivalent

Somewhat Useful

Extremely Useful

## 5. Which of the following workshop components did you find the most useful? (Select up to three)

*The sections described below are not standard across all workshops, and may not have been included in your workshop.

**Hopes and Hesitations:** participants share their expectations for and concerns about the workshop, facilitator discusses how the agenda will address these concerns.

**General Feelings about Pregnancy Options:** participants answer questions around their comfort levels with a patient choosing abortion, adoption, and parenthood in various scenarios. The group discusses which scenarios provoked discomfort.

**The Last Abortion**:participants examine a variety of scenarios where a patient is seeking an abortion and they must grant an abortion to only one.

**Four Corners** (also called “Values Continuum,” or “Values Barometer”): facilitator reads statements and participants physically move along a line representing their level of agreement with the statement.

**Abortion Patient Cases**: group discusses scenarios of challenging patient interactions from provider perspective, as well as patient perspective.

**Challenging Cases**: participants share personal experiences of feeling challenged by patients, and group discusses ways to deal with these situations.

**Personal Challenges**: group discusses feelings about abortion cases they have had or personal challenges in their decisions about providing abortions.

6. What did you like most about the workshop?

Appendix D

7. What did you like least about the workshop?

8. Did the workshop change how you feel about patients that challenge you? If so, how?

9. Can you think of a recent patient interaction in which you would have been able to use the strategies you learned in this workshop if you had had it before the interaction? Please describe.

10. How, if at all, will you use the strategies you practiced in the workshop?


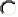

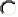


11. As a result of the workshop, did your feelings about caring for women with unintended pregnancy change in any way?

Yes No

**Below you will find 4 scenarios^[[1]](#footnote-1)^ describing different types of patient behaviors. Please read the patient scenario and then indicate your level of agreement with the statements below.**


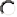

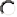

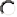

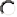

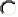

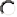

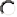

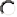

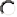

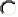

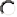

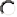

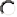

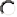

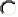


Appendix D

12. A patient who has an undesired pregnancy and chooses to have an abortion.

Strongly disagree

Neutral

Strongly agree

I can think of justifiable reasons that would explain why the patient is in this circumstance and makes this decision.

This case makes me feel frustrated.

My reaction to this case would make it hard for me to care for this patient.


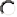

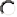

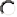

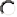

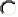

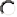

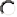

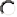

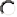

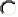

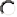

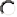

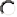

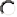

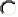


13. A patient who just underwent an abortion and is not interested in starting a birth control method.

Strongly disagree

Neutral

Strongly agree

I can think of justifiable reasons that would explain why the patient is in this circumstance and makes this decision.

This case makes me feel frustrated.

My reaction to this case would make it hard for me to care for this patient.


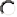

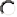

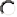

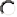

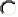

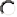

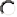

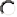

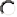

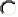

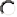

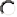

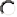

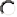

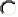


14. A patient who has had an abortion in the past and now presents with an undesired pregnancy and wants an abortion.

Strongly disagree

Neutral

Strongly agree

I can think of justifiable reasons that would explain why the patient is in this circumstance and makes this decision.

This case makes me feel frustrated.

My reaction to this case would make it hard for me to care for this patient.


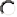

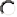

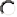

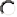

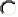

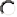

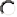

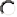

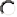

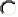

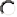

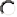

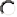

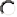

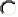


Appendix D

15. A patient who has an undesired pregnancy and presents for care for the first time at 19 weeks’ gestation.

Strongly disagree

Neutral

Strongly agree

I can think of reasons that would explain why the patient is in this circumstance and makes this decision.

This case makes me feel frustrated.

My reaction to this case would make it hard for me to care for this patient.


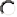

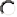

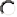

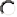

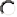

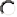

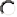

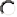

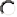

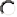

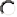

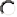

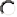

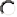

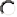

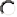

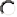

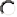

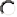

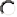

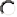

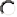

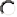

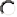

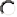

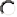

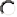

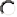

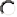

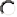

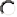

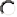

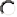


16. Do you intend to directly provide the following procedures in the future -- in training and/or future practice (if your practice allows)?

"Medically indicated" refers to cases for maternal health, fetal anatomic, and genetic anomaly reasons.

[1] [2]

Certainly no Probably no

[3] [4] [5]

Neutral Probably yes Certainly yes

Comprehensive pregnancy options counseling

First-trimester medication abortion for non-medical reasons

First-trimester medication abortion for medically indicated cases

First-trimester uterine aspiration for non-medical reasons

First-trimester uterine aspiration for medically indicated cases

Second-trimester D&E for non-medical reasons

Second-trimester D&E for medically indicated cases

Second-trimester induction for non- medical reasons

Second-trimester induction for medically indicated cases

Referral for abortion care

Disclaimer:

The following question uses a previously validated scale of abortion attitude^[[2]](#footnote-2)^ and may not fully encompass your personal beliefs. Please answer these questions to the best of your ability.

Appendix D

17. The following is a list of reasons a patient may ask for an abortion. Using the given 5-point scale (1= strongly disagree, 5= strongly agree), please indicate whether you agree or disagree that the stated reason is morally acceptable.

[1] Strongly [2] Somewhat disagree disagree

[4] Somewhat [5] Strongly

[3] Neutral agree agree

The patient is financially unable to support the child.

The patient's career/education would be disrupted.

The patient already has too many children.

The pregnancy is a result of rape or incest.

The pregnancy is a threat to the patient's physical health.

18. Please use this space to provide any feedback about the workshop and suggestions on how the workshop can be improved.

1. Case scenarios created by Dr. Jody Steinauer and the Ryan Residency Training Program National Office Staff [↑](#footnote-ref-1)
2. Adapted by authors from: Aiyer AN, Ruiz G, Steinman A, Ho GY. Influence of physician attitudes on willingness to perform abortion. *Obstetrics and gynecology.* 1999;93(4):576-580. [↑](#footnote-ref-2)
