# Supplementary material for: Professionalism in Family Planning Care Workshop
Source: MedEdPORTAL. 2022 Jan 12;18:11212. doi: 10.15766/mep_2374-8265.11212 (PMC8752579; doi:10.15766/mep_2374-8265.11212)
Supplement: Supplementary file 1 — Editable Agendas.docxPFPCW Guide.docxProfessionalism Learner Presurvey.docxProfessionalism Learner Postsurvey.docxProfessionalism Facilitator Postsurvey.docxPFPCW Facilitator Training Video.mp4 [file mep_2374-8265.11212-s001.zip › E. Professionalism Facilitator Postsurvey.docx]

# Ryan Program Professionalism Workshop - Facilitator Post Test

Please complete this post-workshop survey.

This survey will assess your experience facilitating a Professionalism Workshop. The estimated average time to complete the survey is 10 minutes. The data resulting from this evaluation allows us to improve the overall quality of Professionalism Workshops. All information we receive will be handled confidentially. Completed surveys are kept in a password protected database. Any publications or presentations resulting from this study use only aggregate data. No institutions or individuals will be identified.

Your return of a completed survey will indicate your consent to participate in the study. 2020

1. Please enter the date of your presentation (mm/dd/yy)

**Ryan Program Professionalism Workshop - Facilitator Post Test**

2. Please enter your institution name:


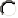

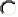


3. Have you taught a workshop previously that discussed abortion or challenging patients, such as a Values Clarification, before?

Yes No

**Ryan Program Professionalism Workshop - Facilitator Post Test**

4. If yes, when, and how many times?

**Ryan Program Professionalism Workshop - Facilitator Post Test**

The rest of this evaluation focuses on the workshop you just facilitated.

5. What kind of learners participated? Please check all that apply.

Medical student Ob-gyn resident

Family medicine resident Resident in other specialty Undergraduate nursing student Physician assistant student Nurse midwifery student Nurse practitioner student Fellow (family planning)

Fellow (non-family planning) Physician

Nurse Midwife

Nurse practitioner Physician assistant Other

6. How many learners / participants attended the workshop?

Note: Please estimate the number of each type of learner to the best of your ability.

Ob-gyn residents

Other residents

Medical students

Other learners

7. What was the setting of the workshop?

(I.e. University hospital, community hospital, ambulatory surgical center, academic setting, Ryan Program, secular hospital, religiously-affiliated institution, etc.)


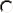

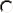

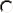

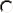

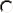


9. How much time did the workshop last?

less than 1 hour 1 - 1.5 hours

1.5 - 2 hours

more than 2 hours Other


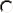

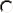

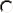

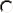

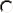


10. How useful do you think the participants found the workshop overall - in terms of giving them some time to reflect on professionalism, challenging cases and abortion care?

Extremely Useless Somewhat Useless Ambivalent Somewhat Useful Extremely Useful


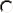

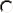

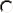

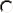

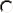

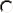

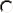

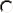

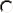

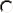

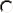

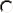

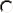

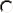

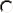

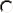

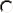

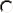

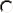

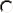


11. Please rate your perceptions of the values of each component of the workshop for the learner.

Extremely Somewhat Somewhat Extremely useless useless Ambivalent useful useful

**Introductions** (where facilitator and participants introduce themselves)

**Ground Rules** (where participants customize and agree to the rules that will govern conduct during the workshop)

**Framing the Conversation** (where the facilitator explains the purpose of the workshop and provides contextual information to inform the discussions and activities that follow)

**Wrap-up** (where participants reflect on what they've learned and have a chance to ask questions)

Appendix E

8. What kind of background materials did you ask them to review?

Articles Videos

Interactive Values Clarification Personal reflection exercises None

Other

## 12. Please rate your perceptions of the values of each component of the workshop for the learner.

Appendix E

(The sections described in question are not standard across all workshops. If you did not facilitate a section described below, please check "This section was not included".)

Introductory Exercise: facilitator and particpants introduce themselves, each person building off commonality with the other

**Hopes and Hesitations:** participants share their expectations for and concerns about the workshop, facilitator discusses how the agenda will address these concerns.

**General Feelings about Pregnancy Options:** participants answer questions around their comfort levels with a patient choosing abortion, adoption, and parenthood in various scenarios. The group discusses which scenarios provoked discomfort.

**The Last Abortion**:participants examine a variety of scenarios where a patient is seeking an abortion and they must grant an abortion to only one.

**Four Corners** (also called “Values Continuum,” or “Values Barometer”): facilitator reads statements and participants physically move along a line representing their level of agreement with the statement.

**Abortion Patient Cases**: group discusses scenarios of challenging patient interactions from provider perspective, as well as patient perspective.

**Challenging Cases**: participants share personal experiences of feeling challenged by patients, and group discusses ways to deal with these situations.

**Personal Challenges**: group discusses feelings about abortion cases they have had or personal challenges in their decisions about providing abortions.

Extremely useless

Somewhat

useless Ambivalent

Somewhat

useful Extremely useful

This section was not included


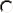

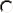

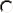

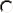

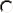

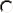

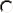

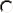

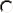

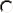

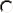

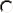

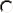

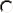

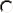

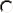

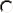

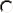

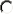

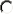

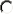

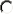

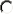

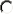

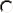

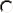

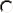

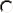

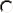

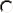

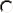

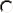

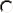

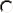

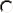

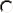

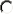

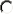

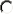

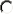

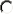

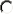

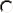

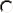

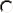

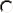

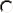

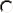

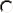

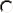

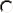

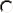

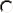

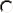


Any other section not listed above

13. If you rated an "other section" in previous question, please describe the additional section you facilitated here. (skip or enter 'NA' if no other section was facilitated.)


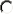

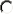

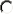

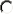


14. How comfortable do you think participants felt sharing their thoughts if they were different from others in the room?

Very comfortable Mostly comfortable Mostly uncomfortable Very uncomfortable


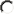

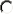

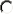

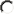

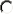

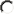

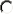

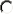


Appendix E

15. How well do you think you did as a faciliator in this workshop? On a scale of 1-10, where 1 indicates not very well and 10 indicates extremely well, please rate how well you think did in the workshop facilitation.

Excellent

Very good

Average

Fair

Poor

Listening to participants while facilitating at the same time

Encouraging participation

Managing time

Managing talkative or quiet participants

Managing participants who make strong or controversial statements

Discussing professionalism

Discussing abortion

Having compassion/ empathy for participants whose beliefs are different than mine

16. *In response to the next few questions, please feel free to write extensive notes. We really appreciate your thoughtfu feedback on the workshop as it will enable us to make improvements.

Please describe the group dynamics in the workshop and how it may have contributed to the overall experience.

17. What aspects of the workshop were most successful?

E

Appendix E

18. What aspects of the workshop do you think were unsuccessful?

19. What were the challenges you might want to address or improve?

20. Which section did you enjoy facilitating the most, and why?

(Possible sections include: Introductions, Ground Rules, Framing the Conversation, Hopes and Hesitations, General Feelings about Pregnancy Options, The Last Abortion, Four Corners, Abortion Patient Cases, Challenging Cases, Personal Challenges, Wrap-up, or other)

21. Which section did you enjoy facilitating the least, and why?

(Possible sections include: Introductions, Ground Rules, Framing the Conversation, Hopes and Hesitations, General Feelings about Pregnancy Options, The Last Abortion, Four Corners, Abortion Patient Cases, Challenging Cases, Personal Challenges, and Wrap-up, or other)

Appendix E

22. What is your relationship to the institution where workshop was held?

Faculty at THIS institution Faculty at other institution

Appendix E

**Ryan Program Professionalism Workshop - Facilitator Post Test**

23. If faculty at this institution, please list any and all leadership roles you hold at this institution.

24. Please use this space to provide any other feedback about the workshop and suggestions on how the workshop can be improved.

25. Please provide your name and date of workshop for our records.

Name

Date of workshop
